# Knowledge and Attitude toward E-Cigarettes among First Year University Students in Riyadh, Saudi Arabia

**DOI:** 10.3390/healthcare11040502

**Published:** 2023-02-08

**Authors:** Shatha A. Alduraywish, Fahad M. Aldakheel, Omar S. Alsuhaibani, Anas D. Bin Jabaan, Rasheed S. Alballa, Ahmad W. Alrashed, Mohammed K. Alhassan, Mohammed K. Aldwaighri

**Affiliations:** 1 Department of Family and Community Medicine, College of Medicine, King Saud University, Riyadh 4545, Saudi Arabia; 2 Department of Clinical Laboratory Sciences, College of Applied Medical Sciences, King Saud University, Riyadh 11433, Saudi Arabia; 3 College of Medicine, King Saud University, Riyadh 11481, Saudi Arabia

**Keywords:** attitude, e-cigarette, knowledge, smoking, university students

## Abstract

Background: Electronic cigarettes are immensely popular among youths across the globe. However, knowledge, attitudes, and perceptions regarding their use vary by country. The present study investigated the knowledge and attitudes toward e-cigarette use among first-year university students in Saudi Arabia. Methods: A cross-sectional design was adopted, and an online, self-administered questionnaire assessing the knowledge of and attitudes toward e-cigarette use was utilized to conduct this study. The study population included students from all streams enrolled in their first year of university. Descriptive statistics were used to report percentages and frequencies, while advanced statistics, such as multiple logistic regression analyses, were used to determine associations. Results: The lifetime and current prevalence of e-cigarette use was 27.4% and 13.5%, respectively, among first-year university students. The mean age of smoking initiation was 16.4 ± 1.2 years. Of e-cigarette users, 31.3% smoked every day and 86.7% used flavored e-cigarettes. Knowledge of the harmful effects of e-cigarettes was high (addiction, 61.2%; asthma, 61%; nicotine content, 75.2%). However, when comparing e-cigarettes to regular cigarettes, only 22.5% and 48.4% of the students reported that they carry the same risk and contain the same chemicals as regular cigarettes do. There was a lack of knowledge (17.1%) regarding government regulations related to e-cigarettes. An attitude of support was observed regarding banning e-cigarettes (2.6 ± 1.5 on a scale of 0 to 4), while at the same time, some associated e-cigarette use with helping to reduce tobacco dependency (2.1 ± 1.2). Marketing adverts were agreed upon to positively influence youth (1.9 ± 1.4). However, the participants’ perceptions relating e-cigarette use to style were not well articulated. Significant gender differences were found: most of the women who participated in the study had better knowledge of e-cigarettes (*p* < 0.001). Being male, having higher income status (OR = 1.67; *p* = 0.013), being a current smoker (OR = 11.6; *p* < 0.001), and having intention for future use (OR = 3.45; *p* < 0.001) were strong predictors of e-cigarette use. Conclusions: These findings suggested the increasing popularity of e-cigarette use among male first-year university students. More educational campaigns and stricter regulations are needed to curb this trend.

## 1. Introduction

Electronic cigarettes (e-cigarettes) have been promoted as a healthier alternative to regular cigarettes since their invention and launch in US markets. From just 7 million users in 2011, the number of e-cigarette users steadily increased to 68 million in 2020 and 82 million adult users in 2021 [1]. Known by a variety of different names, such as “e-cigs”, “e-hookahs”, “vapes”, and “electronic nicotine delivery systems (ENDS)”, e-cigarettes have three types: tanks or mods, rechargeable, and disposable. They comprise a battery, heating element, and e-liquid containing nicotine, certain chemicals, and flavors as the basic elements [2]. The e-cigarette aerosol contains nicotine, which is highly addictive, acetyl compounds as flavoring agents, which are linked to lung damage, cancer-causing chemicals, and heavy metals, such as lead, nickel, and tin [3]. Although e-cigarettes have been regarded as less harmful than conventional cigarettes, they are not harmless. However, long-term studies on the health effects of e-cigarette use have not yet been published. Despite the harmful contents of e-cigarette aerosols, there has been a dramatic increase in the use of e-cigarettes, especially among teens and young adults [4]. Fillipidis et al. examined two-year trends of electronic cigarette use in 27 European countries and demonstrated a significant increase in e-cigarette use from 7% to 11.6%, alongside a simultaneous increase in the perception of harm from 27% to 51.6%, as surveyed from 2012 to 2014 [5]. Generally, there is greater awareness of e-cigarettes in developed countries, although misconceptions and a perceived lack of knowledge has also been found, with claims of e-cigarettes being harmless and a much safer option than conventional tobacco smoking, as well as being an aid to quit tobacco smoking [6,7,8]. The prevalence, trends, and awareness, however, vary by country.

Some studies have associated e-cigarette initiation with younger males wanting to quit tobacco smoking, but this results in the potential risk of these individuals using both and becoming ‘current smokers’ [5,8]. The trends and perceptions around e-cigarette use have been changing, and there are gaps in the existing literature. In addition, the tobacco industry is targeting the vulnerable young adult population and marketing their brands by promoting the quitting of conventional cigarettes amidst uncertainty around the long-term health effects of e-cigarettes. Therefore, it is necessary to examine the current trends and perceptions regarding e-cigarette use in young adults. Although there is literature from the region of Saudi Arabia, most of the research so far has been focused on medical students. Given that adolescents and the young adult population are highly vulnerable, we aimed to investigate the awareness, attitudes, and trends in e-cigarette use in the young Saudi population. The findings may provide data for regulatory bodies that could potentially support stricter regulations.

## 2. Materials and Methods

### 2.1. Study Design and Study Population

An observational analytical cross-sectional study was conducted in order to achieve the study objectives. The primary study site included a large university with many departments, including business, science, engineering, law, and medicine. The study participants primarily included students enrolled in their first year of study and aged between 18 and 24 years. Evidence suggests that smoking among early year university students has risen sharply, either due to peer influence or personality factors [9]. This was the main reason for focusing on first-year students in the study. The study duration was six months, starting from November 2019. A simple random sampling technique was applied to select the study participants from different streams.

Ethical approval was obtained from the Institution’s Review Board Committee prior to the start of the study. IRB Approval No. (E-19-4414).

### 2.2. Sample Size Estimation

According to previously published papers, the reported level of knowledge about e-cigarette was 50% and negative attitudes were 44.9% (15), with a 95% confidence interval and a precision of ±5%, the following equation was used:

$$n=Z^{2}\alpha \;\frac{P\left(1-p\right)}{d^{2}}$$

Z^2^ α = is a standard normal variable (at 5% type 1 error (*p* < 0.05) at 1.96)

*p* = expected proportion in population based on previous studies;

d = precision.

The minimum sample size required was estimated to be 385 participants. With an additional 20% of the number of participants needed to compensate for potential non-responses and incomplete data, the final sample size of the current study was estimated at 462.

### 2.3. Data Collection

The data were collected using a questionnaire comprising four main sections: (i) socio-demographic information, (ii) knowledge assessment, (iii) sources of knowledge, and (iv) measurement of attitude. The first section included information related to age, gender, educational track (health, science, humanities, business, nursing), average household income in the local currency, living conditions, part-time work, and questions regarding tobacco smoking behavior and e-cigarette history. The subsequent sections were adopted from a Malaysian study by Hafiz et al., who validated their questionnaire via their institutional committee [10]. The second section included 11 questions related to the assessment of participants’ knowledge of e-cigarettes. The third section gathered the sources of the participants’ knowledge, and the last section contained 10 questions to assess the attitude of the participants regarding e-cigarettes. The knowledge questions included three responses: “true”, “false”, and “don’t know”. Responses to the attitude questions were given in the forms of “strongly disagree” to “strongly agree” on a five-point Likert scale. The definitions of smoking type included: “ever smokers” or “lifetime prevalence”, namely having ever used cigarettes in their lives, and “current smokers” or “current prevalence”, namely currently using or continuing to use tobacco products.

A pilot study was conducted with 20 subjects to verify the reliability of the tool in the given university setting. These data were not included in the final analysis. Internal consistency was measured on the attitude items using reliability analysis. The Cronbach’s alpha test of reliability was used. The findings showed the 10 items were reliable, with Cronbach’s alpha = 0.81; however, the background factor analysis assessing the factorial validity of the 10 indicators showed that item 32 and item 38 were not reliable due to poor item factor total loadings. As such, they were excluded from the total attitude score.

Informed consent was obtained from all participants who wished to participate in the study prior to the start of the data collection. Confidentiality of data was assured.

### 2.4. Study Variables and Data Collection

The study intended to measure knowledge and attitudes towards e-cigarettes as major dependent outcome variables. Independent variables included sociodemographic factors. The number of students enrolled in the first year of each stream was obtained and proportional probability sampling was applied based on the sample size. Sample selection was based on a multistage simple random sampling technique of students in the chosen colleges. First, the study stream/college was chosen, then the first-year class roll was obtained to determine the number of students, and then the students were randomly selected. The purpose of the study was explained, and the student was enrolled if they agreed to participate in the study. Anonymity was maintained and consent was obtained prior to the start of data collection. The questionnaire was self-administered online using Google Docs.

### 2.5. Data Analysis

Statistical analysis was conducted using IBM SPSS version 25.0. Frequencies and percentages were used to describe categorical variables, while continuous variables were reported by the mean and standard deviation. The students’ total knowledge score was computed based on correctly answered questions, and the attitude score was derived based on Likert-scale points. The univariate non-parametric chi-square test was used to assess the statistical significance of students’ responses to the e-cigarette knowledge indicators. The bivariate chi-square test of association was used to assess the correlation between categorical variables, and the independent samples *t*-test was used to compare the mean scores between the levels of binary variables. Multivariate binary logistic regression analysis was used to assess the significant predictors of students’ past and current use of e-cigarettes. In the secondary analysis, the association between the students’ sociodemographic factors and level of knowledge and their perceived attitudes toward e-cigarettes as a dependent outcome variable was examined. A *p*-value of ≤0.05 was considered statistically significant.

## 3. Results

### 3.1. Participants’ Socio-Demographic Characteristics

The socio-demographic characteristics of the study participants are displayed in Table 1. The total study sample included 467 completed responses. The mean age of the participants was 18.6 ± 0.7 years. Most of the study participants were male (52.2%), and the majority reported a monthly household income greater than SAR 10,000 (77.8%). Most of the participants were enrolled in the top three study tracks, namely science (47%), health (25.7%), and business (11.8%). Around 14% of the students were ever smokers (current and former smokers). Around 61.5% of the current smokers usually used single-type tobacco. Of the study participants, 27.4% were e-cigarette ever-users, of whom 65 (50.7%) were former users. Of the e-cigarette users, 31.3% smoked every day and 86.7% used flavored e-cigarettes.

### 3.2. Knowledge about E-Cigarettes

Almost the entire study population (99%) was aware of e-cigarettes. Generally, knowledge related to the harmful effects of e-cigarettes was higher (addiction, 61.2%; allergens and asthma, 61%; nicotine content, 75.2%). When comparing e-cigarettes to regular cigarettes, 22.5% and 48.4% of the students agreed that they carry the same risk and have the same chemicals as normal cigarettes. However, there was a lack of knowledge (17.1%) regarding government regulations related to e-cigarettes. Other details related to overall e-cigarette knowledge are shown in Table 2.

Furthermore, responses to knowledge items according to gender and smoking status are shown in Table 3. Significant differences between men and women were found in the perception of the harmful effects of e-cigarettes.

A comparison of knowledge according to smoking status showed that 63.6% of the non-smokers believed that e-cigarettes are addictive, compared to 47% of those who had ever smoked (*p* < 0.001). Additionally, 59.1% of those who were ever-smokers thought e-cigarettes were less harmful to health than traditional cigarettes, compared to 35.9% of those who never smoked (*p* < 0.001). In general, most of the non-smokers (86%) correctly identified the misleading statement that e-cigarettes are not harmful to health as wrong when compared to those who had ever smoked (69.7%) (*p* < 0.001).

### 3.3. Sources of E-Cigarette Information

**Figure 1:**
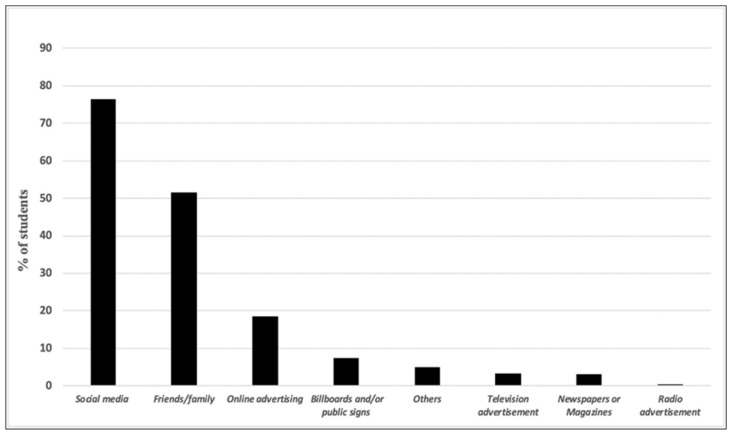
Source of knowledge regarding e-cigarettes among first-year university students.

Sources of knowledge regarding e-cigarettes information are displayed in Figure 1. Social media (76.4%) played a predominant role as a medium for learning information about e-cigarettes, followed by family and friends (51.6%) and online advertising content (18.4%).

### 3.4. Attitudes toward E-Cigarettes

The attitudes of students towards e-cigarettes was assessed and are displayed in Table 4. Strong disagreement was expressed in a way that suggested a negative attitude towards e-cigarettes. Most disagreed that e-cigarettes are fun (61.9%), that they make someone look stylish (84.6%), or that they increase one’s concentration (71.3%), while more than half of the respondents disagreed that e-cigarettes relieve stress (77.5%). On the other hand, 39% of participants agreed that e-cigarettes help to cut down tobacco smoking.

Furthermore, the attitudes of the participants towards e-cigarette use were assessed and analyzed based on gender (Table 5). Significant gender differences were found: more women expressed disapproval. Most of the women disagreed with using e-cigarettes for fun (73.6% vs. 51.2%; *p* < 0.001), for style (91% vs. 52.4%; *p* < 0.001), and that e-cigarettes help relieve stress (60.6% vs. 44.2%; *p* < 0.001) and improves one’s image (91.5% vs. 82.4; *p* < 0.001). More men agreed that e-cigarettes help reduce tobacco smoking than women (20.7% vs. 55.7%; *p* < 0.001).

### 3.5. Predictors of E-Cigarette Use

The predictors of e-cigarette use in the overall study population are presented in Table 6. Being male, having higher income status, and being current smokers were strong predictors of e-cigarette use. Students with a household income greater than SAR 10,000 were at 1.67 times (*p* = 0.013) more at risk of using e-cigarettes than lower income students. Furthermore, the intention to use e-cigarettes in the future increased the odds that they would by 3.45 times (*p* < 0.001), suggesting that the students favored e-cigarette use. Being a smoker carried the highest odds of being 11.6 times (*p* < 0.001) more likely to use e-cigarettes than non-smokers. Female gender generally showed that a protective effect (OR= 0.42; *p* = 0.89) with a negative attitude and strong disagreement towards e-cigarettes was more prevalent among women.

## 4. Discussion

This study was conducted to investigate the knowledge and attitudes of newly enrolled university students toward e-cigarette use. According to the present study, the lifetime and current prevalence of e-cigarette use was 27.4% and 13.5% among first-year university students. The popularity of flavored cigarettes was very high, and one-third of the current users used e-cigarettes every day. Around 16% of the study population demonstrated a positive attitude toward future e-cigarette use. The present study showed relatively moderate awareness of the harmful effects of e-cigarette use, and half of the study population thought that e-cigarettes and conventional cigarettes have the same composition. However, there was low awareness of government regulations.

On comparing the prevalence of e-cigarette use with data from the Southeast Asian region, our study showed a higher prevalence. The lifetime and current prevalence in a study from South Korea showed 11% and 2%, respectively [11]; 2.1% in Chinese participants [7]; and 4.3% current prevalence in Japanese adolescents [12]. On the other hand, a systematic review of the global prevalence of e-cigarette use in America, Europe, and Asia reported current prevalence at 10%, 14%, and 11% [4]. Another recent study, in an effort to undertake a global tobacco evaluation, examined the current use of e-cigarettes in 14 countries among people older than 15 years. The current prevalence of e-cigarette use ranged from 0.02% to 3.5%, reported in ascending order as follows: India (0.02%), Bangladesh (0.2%), China (0.9%), Costa Rica and Turkey (1.3%), Ukraine (1.7%), Romania (3.4%), and Russia (3.5%) [13]. However, the reason for the lower prevalence overall can be attributed to the inclusion of a younger population. The young adult population may generally show higher figures than the general population.

Furthermore, studies from the Saudi Arabian region have reported a current prevalence of 10% and 12% among medical students from different universities in the central region [14,15]. These results were consistent with ours, although the present study showed a higher rate. On the contrary, regarding e-cigarette use, studies from the western region of Saudi Arabia showed higher prevalence rates of 27% and 21% in medical universities in Jeddah and Jazan, respectively [16,17]. The findings suggest an increasing prevalence of e-cigarette use among young Saudi adults compared to other regions globally.

Our findings showed that most of the respondents were well aware of the addictive effects and the presence of nicotine in e-cigarettes. However, they had low awareness regarding the chemical composition of e-cigarettes and were not sure of the government regulations relating to them. In addition, they considered e-cigarettes to be less harmful than conventional cigarettes. Furthermore, more female students and non-smokers reported e-cigarettes to be harmful and addictive, with more health risks compared to traditional cigarettes, and described the e-cigarette vapor as dangerous to children. These findings suggest that men who smoke showed a decline in their harm perception of e-cigarettes. This was further supported by the negative outlook predominantly stated by female participants, who disapproved of e-cigarette use for enhancing performance or for style and image. Our findings were consistent with other similar studies. King et al. and Hughes et al. reported lower harm perception among male smokers in American and British youths [18,19]. Regional studies from Saudi Arabia also showed similar results [20].

Another important finding that is of note is the students’ perception of e-cigarette use as a tool for reducing tobacco dependency. Previous research has generated mixed results on perceptions of e-cigarette use as a tool to reduce tobacco dependency. Some studies have demonstrated support for our findings, while others are in stark contradiction. Data from a medical school in the United States reported that males who have tried tobacco smoking usually support the use of e-cigarettes for smoking cessation, while non-smokers did not favor this view [21]. Almutham also demonstrated similar findings, expressing disagreement regarding considering e-cigarettes as an alternative to traditional smoking [14]. This remains a subject of uncertainty and dispute, since substantial evidence has not yet been generated highlighting the risks versus benefits of e-cigarette use in smoking cessation. Moreover, students’ perceptions are also influenced by marketing claims promoting e-cigarettes as healthy, generating biased thinking.

In addition, a strong attitude towards banning e-cigarettes was expressed in the current study. These findings suggest that most students are in favor of educational programs and anti-smoking campaigns.

A significant association of e-cigarette use with male gender, current smokers, and affluent status was an important finding of this study. In addition, a strong positive attitude toward e-cigarette use and intention for future use were also found to be associated with e-cigarette use. Female gender showed a protective effect against e-cigarette use. Higher-income status and current smoking have been shown to influence e-cigarette use in other global studies, but the influence of gender remains disputed [13,18]. The conservative nature of Saudi Arabia may be a reason for our findings regarding gender differences. Further studies are recommended to explore the changing trends in gender influence and perception toward future use. Moreover, in the present study, perceptions were mostly influenced by social media. Social media can also serve as a powerful platform to generate and propagate information in mass campaigning efforts.

The rapid rise in the popularity of e-cigarettes and increased marketing have influenced adolescents and youth globally to experiment with e-cigarettes. The early age of initiation is another point of concern, since the present study found early initiation of cigarette use among adolescents and children. These findings point toward an increasing need for regulatory actions, not only for adults but also to include curbing strategies for school children as well.

The present study has certain limitations. The study has limited generalizability as the study population was restricted in terms of age and region. The sample was attained from a single center. The risk of bias also cannot be eliminated due to the self-administrated survey. Qualitative studies with in-depth research are warranted to understand youth perception of e-cigarette use and to determine the factors associated with initiation.

## 5. Conclusions

To conclude, our study updated the existing literature on e-cigarette use. The associated factors, age of smoking initiation, and intention for future use must be noted when addressing e-cigarette trends. Health education campaigns in schools and on university campuses are needed. Addressing the debate on the safety of e-cigarettes over conventional cigarettes is also essential.

## Figures and Tables

**Table 1:** Participants’ socio-demographic characteristics and smoking history.

Participants’ Characteristics	N = 467 *n* (%)
Gender	
Male Female	244 (52.2) 233 (47.8)
Age in years (Mean ± SD)	18.6 ± 0.7
Living conditions	
House Apartment Compound	413 (88.4) 35 (7.5) 19 (4.1)
Part time job	
Yes No	42 (9.0) 425 (91.0)
Approximate household monthly income (SR *)	
≤5000 5001 to ≤ 10,000 10,001 to ≤ 20,000 >20,000	24 (5.1) 80 (17.1) 160 (34.3) 203 (43.5)
Tracks **	
Health Science Humanities Business Nursing	120 (25.7) 220 (47.1) 40 (8.6) 55 (11.8) 32 (6.9)
Streams	
Medical Non-medical	152 (32.5) 315 (67.5)
Ever smoking (including e-cigarettes)	
Yes No	66 (14.1) 401 (85.9)
Mean age at smoking initiation (Mean ± SD) in years	16.3 (1.2)
Smoking status	
Current smoker Former smoker Never smoked	52 (11.1) 14 (3.0) 401 (85.9)
Types of tobacco smoked ***	
One type More than one type	40 (61.5) 12 (38.5)
Types of tobacco used ***	
Cigarettes Cigar Roll your own (RYO) Shisha (water pipe) Other	50 (75.7) 10 (15.4) 2 (3.1) 30 (45.4) 7 (10.6)
Ever used e-cigarettes	
Yes No	128 (27.4) 339 (72.6)
E-cigarette usage status	
Former user Current user	65 (50.7) 63 (49.2)
Use of flavored e-cigarettes	
Yes No	111 (86.7) 17 (13.3)

* SAR (Saudi Arabian Riyal); ** Based on the KSU Track System; *** For current smokers only.

**Table 2:** Overall knowledge regarding e-cigarettes among first-year university students (N = 467).

Knowledge Items	Participants’ Responses			
	True *n (%)*	False *n (%)*	Don’t Know *n (%)*	*p*-Value *
1. Have you ever heard about e-cigarettes?	462 (99)	4 (0.9)	1 (0.2)	<0.001
2. E-cigarettes are addictive.	286 (61.2)	63 (13.5)	118 (25.3)	<0.001
3. E-cigarettes are a potential cause of asthma attacks and allergies.	285 (61)	26 (5.6)	156 (33.4)	<0.001
4. E-cigarettes can contain nicotine.	351 (75.2)	15 (3.2)	101 (21.6)	<0.001
5. The health risks of e-cigarette are the same as normal cigarettes.	105 (22.5)	232 (49.7)	130 (27.8)	<0.001
6. E-cigarettes have the same chemicals as normal cigarettes.	226 (48.4)	92 (19.7)	149 (31.9)	<0.001
7. Are you aware of any regulations by the government on e-cigarettes?	80 (17.1)	232 (49.7)	155 (33.2)	0.005
8. E-cigarettes are less harmful to health than normal cigarettes.	183 (39.2)	158 (33.8)	126 (27)	<0.001
9. E-cigarettes are not harmful to health.	30 (6.4)	390 (83.5)	47 (10.1)	<0.001
10. Can e-cigarettes be used in smoke-free places?	56 (12)	313 (67)	98 (21)	<0.001
11. E-cigarette vapor is dangerous to babies and children.	378 (80.9)	17 (3.6)	72 (15.4)	<0.001

* *p*-value of chi-squared univariate goodness-of-fit test.

**Table 3:** Knowledge regarding e-cigarettes among first-year university students according to gender and smoking status.

Knowledge Item	Participants’ Responses													
	Male N = 244 *n* (%)			Female N = 223 *n* (%)			*p*-Value	Non-Smokers N = 401 *n* (%)			Ever Smoked * N = 66 *n* (%)			*p*-Value
	Yes	No	I Do Not Know	Yes	No	I Do Not Know		Yes	No	I Do Not Know	Yes	No	I Do Not Know	
1. Have you ever heard about e-cigarettes?	242 (99.2)	1 (0.4)	1 (0.4)	220 (98.7)	3 (1.3)	0 (0.0)	<0.001	398 (99.3)	3 (0.7)	0 (0)	64 (97.0)	1 (1.5)	1 (1.5)	<0.001
2. E-cigarettes are addictive.	136 (55.7)	50 (20.5)	58 (23.8)	150 (67.3)	13 (5.8)	60 (26.9)	<0.001	255 (63.6)	35 (8.7)	111 (27.7)	31 (47.0)	28 (42.4)	7 (10.6)	<0.001
3. E-cigarettes are a potential cause of asthma attacks and allergies.	122 (50.0)	22 (9.0)	100 (41.0)	163 (73.1)	4 (1.8)	56 (25.1)	<0.001	262 (65.3)	11 (2.7)	128 (31.9)	23 (34.8)	15 (22.7)	28 (42.4)	0.142
4. E-cigarettes can contain nicotine.	191 (78.3)	11 (4.5)	42 (17.2)	160 (71.7)	4 (1.8)	59 (26.5)	<0.001	292 (72.8)	13 (3.2)	96 (23.9)	59 (89.4)	2 (3.0)	5 (7.6)	<0.001
5. The health risks of e-cigarette are the same as traditional cigarettes.	50 (20.5)	136 (55.7)	58 (23.8)	55 (24.7)	96 (43.0)	72 (32.3)	0.003	89 (22.2)	194 (48.4)	118 (29.4)	16 (24.2)	38 (57.6)	12 (18.2)	<0.001
6. E-cigarettes have the same chemicals as traditional cigarettes.	103 (42.2)	66 (27.0)	75 (30.7)	123 (55.2)	26 (11.7)	74 (33.2)	<0.001	202 (50.4)	65 (16.2)	134 (33.4)	24 (36.4)	27 (40.9)	15 (22.7)	0.170
7. Are you aware of any regulations by the government on e-cigarettes?	47 (19.3)	108 (44.3)	89 (36.5)	33 (14.8)	124 (55.6)	66 (29.6)	<0.001	64 (16.0)	205 (51.1)	132 (32.9)	16 (24.2)	27 (40.9)	23 (34.8)	0.244
8. E-cigarettes are less harmful to health than traditional cigarettes.	120 (49.2)	56 (23.0)	68 (27.9)	63 (28.3)	102 (45.7)	58 (26.0)	<0.001	144 (35.9)	143 (35.7)	114 (28.4)	39 (59.1)	15 (22.7)	12 (18.2)	<0.001
9. E-cigarettes are not harmful to health.	20 (8.2)	194 (79.5)	30 (12.3)	10 (4.5)	196 (87.9)	17 (7.6)	<0.001	25 (6.2)	344 (85.8)	32 (8.0)	5 (7.6)	46 (69.7)	15 (22.7)	<0.001
10. Can e-cigarettes be used in smoke-free places?	40 (16.4)	159 (65.2)	45 (18.4)	16 (7.2)	154 (69.1)	53 (23.8)	<0.001	40 (10.0)	275 (68.6)	86 (21.4)	16 (24.2)	38 (57.6)	12 (18.2)	<0.001
11. E-cigarette vapor is dangerous to babies and children.	12 (4.9)	186 (76.2)	46 (18.9)	192 (86.1)	5 (2.2)	26 (11.7)	<0.001	329 (82.0)	13 (3.2)	59 (14.7)	49 (74.2)	4 (6.1)	13 (19.7)	<0.001

* Includes current and former smokers.

**Table 4:** Overall attitude towards e-cigarettes among first-year university students.

Attitudes Items	Participants’ Responses N = 467 *n* (%)					*p* Value
	SA	A	NS	D	SD	
Using e-cigarettes is fun.	45 (9.6)	55 (11.8)	78 (16.7)	89 (19.1)	200 (42.8)	<0.001
E-cigarettes make someone look stylish.	19 (4.1)	23 (4.9)	30 (6.4)	120 (25.7)	275 (58.9)	<0.001
E-cigarettes relieve one’s stress.	27 (5.8)	50 (10.7)	147 (31.5)	104 (22.3)	139 (29.8)	<0.001
E-cigarettes have a problem-solving effect.	24 (5.1)	21 (4.5)	60 (12.8)	140 (30)	222 (47.5)	<0.001
E-cigarettes help to cut down on tobacco smoking.	91 (19.5)	91 (19.5)	164 (35.1)	53 (11.3)	68 (14.6)	<0.001
E-cigarettes enhance one’s performance.	19 (4.1)	21 (4.5)	72 (15.4)	128 (27.4)	227 (48.6)	<0.001
E-cigarettes increase one’s concentration.	17 (3.6)	20 (4.3)	97 (20.8)	127 (27.2)	206 (44.1)	<0.001
E-cigarettes improve one’s image.	15 (3.2)	15 (3.2)	32 (6.9)	133 (28.5)	272 (58.2)	<0.001

SA = strongly agree; A = agree; NS = not sure; D = disagree; SD = strongly disagree.

**Table 5:** Attitude towards e-cigarettes among first-year university students, stratified by gender.

Attitudes Items	Participants’ Responses N = 467										
	Male (N = 244) *n* (%)					Female (N = 223) *n* (%)					
	SA	A	NS	D	SD	SA	A	NS	D	SD	*p* Value
Using e-cigarettes is fun.	34 (13.9)	40 (16.4)	45 (18.4)	40 (16.4)	85 (34.8)	11 (4.9)	15 (6.7)	33 (14.8)	49 (22.0)	115 (51.6)	<0.001
E-cigarettes make someone look stylish.	15 (6.1)	18 (7.4)	19 (7.8)	64 (26.2)	128 (26.2)	4 (1.8)	5 (2.2)	11 (4.9)	56 (25.1)	147 (65.9)	<0.001
E-cigarettes relieve one’s stress.	21 (8.6)	34 (13.9)	81 (33.2)	45 (18.4)	63 (25.8)	6 (2.7)	16 (7.2)	66 (29.6)	59 (26.5)	76 (34.1)	<0.001
E-cigarettes have a problem-solving effect.	18 (7.4)	17 (7.0)	30 (12.3)	79 (32.4)	100 (41.0)	6 (2.7)	4 (1.8)	30 (13.5)	61 (27.4)	122 (54.7)	<0.001
E-cigarettes help to cut down on tobacco smoking.	75 (30.7)	61 (25.0)	65 (26.6)	16 (6.6)	27 (11.1)	16 (7.2)	30 (13.5)	99 (44.4)	37 (16.6)	41 (18.4)	<0.001
E-cigarettes enhance one’s performance.	16 (6.6)	16 (6.6)	42 (17.2)	67 (27.5)	103 (42.2)	3 (1.3)	5 (2.2)	30 (13.5)	61 (27.4)	124 (55.6)	<0.001
E-cigarettes increase one’s concentration.	15 (6.1)	17 (7.0)	50 (20.5)	68 (27.9)	94 (38.5)	2 (0.9)	3 (1.3)	47 (21.1)	59 (26.5)	112 (50.2)	<0.001
E-cigarettes improve one’s image.	12 (4.9)	11 (4.5)	20 (8.2)	73 (29.9)	128 (52.5)	3 (1.3)	4 (1.8)	12 (5.4)	60 (26.9)	144 (64.6)	<0.001

SA = strongly agree; A = agree; NS = not sure; D = disagree; SD = strongly disagree.

**Table 6:** Predictors of e-cigarette use by multivariate logistic regression analysis.

Predictors	Adjusted Odds Ratio	95% CI		*p*-Value
		Lower	Upper	
Age of the student	0.94	0.57	1.5	0.815
Sex of the student	0.42	0.21	0.84	**0.014**
Study track	1.0	0.75	1.3	0.896
Household monthly income (SAR): income >10,000	1.6	1.11	2.5	**0.013**
Living conditions	1.5	0.86	2.8	0.140
Part time job = Yes	1.5	0.54	4.4	0.407
Current smoker = Yes	11.6	4.5	29.4	**<0.001**
Knowledge regarding e-cigarettes	1.1	0.98	1.2	0.087
Attitudes toward e-cigarettes	1.1	1.1	1.1	**<0.001**
Intention to use e-cigarettes next year	3.5	2.3	5.3	**<0.001**
Constant	0.02			0.39

Dependent variable = e-cigarette use (0 = No/1 = Yes). The bold indicates statistical significance.

## Data Availability

Not applicable.
